# Cannabis and nicotine use are independently associated with adverse surgical, medical, and psychosocial outcomes following upper extremity fracture fixation

**DOI:** 10.1186/s13018-025-06635-w

**Published:** 2026-01-19

**Authors:** Christopher D. Hamad, Nora A. Galoustian, Joshua Wiener, Nick Yusin, Timothy Liu, Thomas Olson, Paul Walker, Soroush Shahamatdar, Michelle Nwufo, Autreen Golzar, David C. Kaelber, Nicholas M. Bernthal, Christopher Lee, William L. Sheppard

**Affiliations:** 1 Department of Orthopaedic Surgery, University of California, Los Angeles, CA USA; 2 David Geffen School of Medicine at UCLA, Los Angeles, CA USA; 3 Case Western Reserve University School of Medicine, Cleveland, OH USA; 4 University of Southern California, Los Angeles, CA USA; 5 Population and Quantitative Health Sciences, Case Western Reserve University and The Center for Clinical Informatics Research and Education, The MetroHealth System, Cleveland, OH USA; 6 Department of Orthopaedic Surgery, UCLA Santa Monica Medical Center, 1225 15th Street, Suite 3144B, Santa Monica, CA 90404 USA

**Keywords:** Cannabis, Nicotine, Upper extremity fractures, Orthopaedic trauma, Postoperative complications, Propensity score matching

## Abstract

**Background:**

Marijuana use is rising in the United States, yet its impact on perioperative outcomes remains poorly understood, particularly in orthopaedic trauma where cessation is often not feasible. This study evaluates the risks associated with cannabis and nicotine use in patients undergoing fixation of upper extremity fractures.

**Methods:**

We performed a retrospective analysis of adult trauma patients with upper extremity fractures (2015–2023) identified using CPT codes for surgical fixation in the TriNetX database. Four cohorts were defined: cannabis-only users (*n* = 801), nicotine-only users (*n* = 14,310), concurrent users (*n* = 901), and non-users matched 1:1 to each exposure cohort. Propensity score matching was applied to each pairwise comparison. Primary outcomes were surgical and medical complications; secondary outcomes included new postoperative psychosocial diagnoses (anxiety, depression, opioid use disorder, and chronic pain) and coagulation parameters. Binary outcomes were compared using absolute risk differences, risk ratios, odds ratios, and 95% confidence intervals; continuous outcomes with independent t-tests, all assessed within 1 year following surgery.

**Results:**

Cannabis-only users had significantly higher rates of implant-related infection, reoperation, readmission, depression, and anxiety compared with non-users (*p* < 0.05). Nicotine-only users demonstrated higher odds ratios in most overlapping outcomes and showed significantly elevated rates across a broader range of complications, including superficial and deep infection, nonunion or malunion, wound dehiscence, pneumonia, chronic pain, mortality, and psychosocial complications. Concurrent users did not demonstrate additive risk compared with cannabis-only users.

**Conclusion:**

Cannabis and nicotine use were independently associated with increased postoperative complications following fixation of upper extremity fractures compared with matched non-user controls. The absence of statistically significant additive effects may reflect limited power to detect modest interactions, overlapping biological mechanisms, or a true absence of synergy. These findings support standardized screening, risk stratification, and targeted perioperative strategies, including extended antibiotic prophylaxis and integrated psychosocial support, to reduce complications in this at-risk population.

**Supplementary Information:**

The online version contains supplementary material available at 10.1186/s13018-025-06635-w.

## Introduction

Marijuana use is steadily rising in the United States, with approximately one in five Americans reporting recent use [1]. Prevalence is highest among young adult males between 21 and 34 years of age, a demographic that also represents the highest risk group for sustaining orthopaedic trauma injuries [2–5]. Following the legalization of recreational marijuana in Colorado and Washington in 2012, a nationwide trend ensued, with 25 states enacting similar laws by 2024 [1–3]. As legalization and societal acceptance grow, daily cannabis use has become increasingly common [1]. Despite this trend, the perioperative risks of cannabis remain poorly understood, particularly in orthopaedic trauma surgery where immediate cessation after injury may not significantly diminish the impact of this drug.

Concerns about cannabis use in the perioperative period are supported by in vitro and animal studies demonstrating adverse effects on both immune and bony physiology. Tetrahydrocannabinol (THC), the primary psychoactive component of cannabis, impairs immune cell recruitment, alters cytokine signaling, and suppresses osteogenesis [4–6]. These immunomodulatory effects may impair fracture healing and compromise local antimicrobial defenses, potentially elevating the risk of implant-related infection or delayed union [7]. 

Clinical data, however, remain mixed. In pediatric patients, cannabis use is associated with delayed union [8]. In adult tibial shaft fractures, cannabis users show initially elevated risks of deep infection and surgical complications, although these associations lost significance after multivariate adjustment [9]. Two retrospective studies on distal radius fractures report higher infection and malunion rates in cannabis users, particularly among those who also used tobacco, with additional associations noted for medical complications, nerve injuries, and tendon injuries [10, 11]. Among patients undergoing ankle open reduction internal fixation (ORIF), combined cannabis and tobacco use is linked to higher rates of urinary tract infections, readmissions, and adverse events, while cannabis-only users show no increased risk [12]. Cannabis use is also associated with higher rates of scaphoid nonunion and subsequent revision surgery at 6, 12, and 24 months [13–17]. Additionally, thromboelastography (TEG) analysis in trauma patients who use cannabis demonstrates prolonged reaction times (R-time), indicating delayed clot initiation and underlying coagulopathy that may confer increased risk perioperatively [18]. Cannabis-associated coagulopathy may be mediated through cannabinoid receptor pathways that inhibit platelet activation and aggregation. Experimental studies show impaired glycoprotein VI signaling, reduced alpha-granule release, decreased fibrinogen binding, and attenuation of thrombin-induced clot formation [19–21]. 

In parallel, nicotine use alone is a well-established risk factor for adverse postoperative outcomes, including impaired wound healing, infection, venous thromboembolism, and pulmonary complications [12]. Despite this, limited data exist regarding the potential additive risk of combined cannabis and nicotine use in upper extremity fracture surgery, specifically regarding implant-related infection and coagulopathy following ORIF [10]. 

Psychosocial morbidity is also highly prevalent following orthopaedic trauma. Depression and post-traumatic stress disorder (PTSD) with a recent systematic review reporting weighted pooled prevalences of 32.6% and 26.6%, respectively [22]. Anxiety disorders are similarly common, with generalized anxiety disorder occurring in approximately 10–14% of patients depending on injury severity and anatomical region [23]. Given this substantial baseline vulnerability, even modest increases in psychosocial complications among cannabis-using trauma patients may have meaningful implications for recovery, readmission risk, and long-term functional outcomes.

This retrospective cohort study aims to identify postoperative complications associated with cannabis and combined cannabis-nicotine use following fixation of upper extremity fractures, using a large, multi-institutional dataset drawn from U.S. healthcare organizations. Additionally, we evaluate differences in coagulation markers, prothrombin time (PT) and activated partial thromboplastin time (aPTT), to further characterize thrombotic risk. All postoperative surgical, medical, psychosocial, and laboratory-based complications were assessed within a standardized 1-year follow-up period after fracture fixation to allow consistent comparison across exposure groups. Findings from this study may enhance preoperative risk stratification, inform perioperative management, and guide interventions to reduce complications in an increasingly prevalent subset of orthopedic trauma patients.

## Methods

### Data source and study design

We conducted a retrospective cohort study using the TriNetX Research Network (Cambridge, MA; www.trinetx.com), a federated health research platform aggregating de-identified electronic health records from 92 healthcare organizations across the United States with over 130 million patients. The platform provides real-time access to structured clinical data, including demographics, diagnoses, procedures, medications, and laboratory results. Data access was supported by the National Center for Advancing Translational Sciences (NCATS) grant UM1TR004528.

Data were accessed through the TriNetX Research Network on August 12, 2025, and final analyses were subsequently performed. All data within TriNetX are de-identified in accordance with the Health Insurance Portability and Accountability Act (HIPAA) Privacy Rule under 45 CFR § 164.514(b)(1), utilizing expert-determined methods such as obfuscation and minimum cell sizes (≥ 10 individuals per value). Therefore, the dataset does not constitute protected health information and is exempt from HIPAA and IRB oversight.

### Cohort definitions

**Fig. 1:**
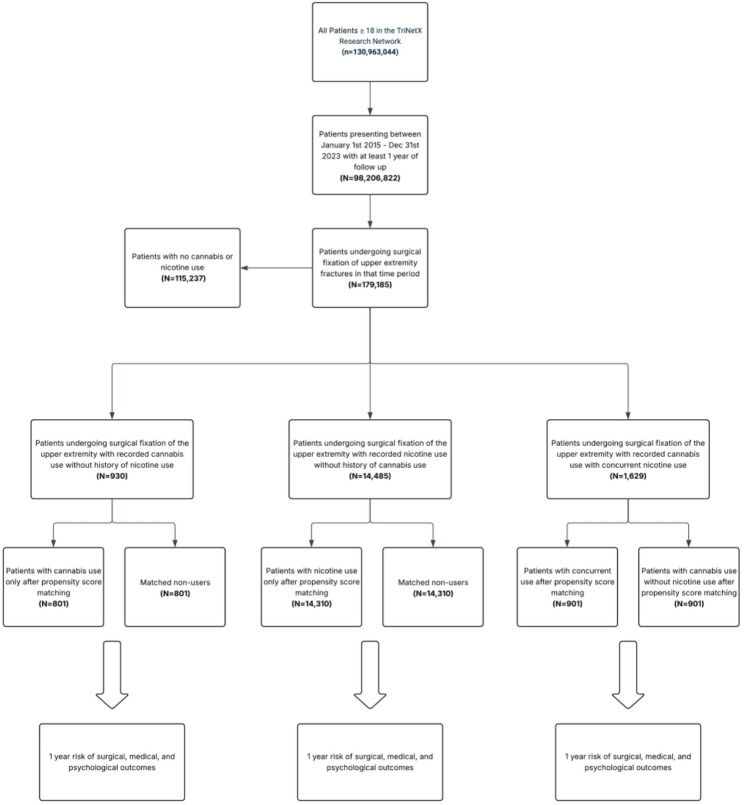
Cohort selection diagram

We identified adult patients (aged ≥ 18 years) who underwent surgical fixation of upper extremity fractures between January 1, 2015, and December 31, 2023. We limited the analysis to upper extremity injuries because they involve non-weight-bearing extremities and have distinct postoperative mobility and rehabilitation patterns compared with lower extremity fractures. Eligible procedures were defined using a comprehensive list of Current Procedural Terminology (CPT) codes for surgical fixation involving the scapula, shoulder, humerus, elbow, radius, ulna, wrist, and hand. To ensure transparency and reproducibility, all CPT codes were selected a priori to comprehensively capture all upper extremity ORIF procedures based on standard orthopedic operative categories; full code list provided in Table S1. Patients were included at the patient level rather than the fracture level; therefore, individuals with multiple upper extremity fractures were counted once based on their first qualifying fixation procedure. Patients with concomitant lower extremity injuries were not excluded, but these injuries were accounted for as covariates during propensity score matching to mitigate confounding from unrelated trauma and mobility limitations. Patients were then stratified into three exposure cohorts based on International Classification of Diseases, Tenth Revision (ICD-10) diagnostic codes for substance use. Cannabis-only users included patients with a diagnosis of cannabis-related disorders (F12) and no diagnosis of nicotine dependence (F17), with cannabis use documented at least one day prior to surgery. Non-users included patients without documentation of either cannabis-related disorders or nicotine dependence. The concurrent users cohort comprised patients with documented diagnoses of both cannabis-related disorders and nicotine dependence. Each pairwise comparison (cannabis-only users vs. non-users, cannabis-only users vs. concurrent users, and nicotine-only users vs. non-users) was analyzed independently to assess the differential risk associated with cannabis and/or nicotine exposure. Figure 1 outlines the cohort selection process.

### Index event and follow-up

The index event was defined as the first qualifying upper extremity fracture surgery within the study period. Exact injury dates are not uniformly available within TriNetX. To maintain temporal validity, we required that all fracture diagnoses establishing cohort eligibility be recorded on or prior to the date of fixation, ensuring that the injury clearly preceded the index surgical event. Follow-up began one day after the index event and continued for 365 days. To ensure data reliability, patients whose index events occurred 20 or more years prior to the query date were excluded, although no patients met this criterion.

### Outcomes

Primary and secondary outcomes included a range of medical, surgical, and psychosocial complications commonly evaluated in orthopedic trauma and substance use literature. Outcomes were defined using ICD-10 and CPT codes mapped through TriNetX’s Unified Medical Language System (UMLS) ontology. Clinical endpoints included wound dehiscence, superficial wound infection, implant-related infection, nonunion or malunion, nerve palsy, irrigation and debridement, amputation, reoperation, blood transfusion, pulmonary embolism (PE), deep vein thrombosis (DVT), pneumonia, acute respiratory distress syndrome (ARDS), myocardial infarction (MI), stroke, mortality, anxiety, depression, opioid use disorder, chronic pain, and all-cause readmission. Laboratory-based outcomes included prothrombin time (PT) and activated partial thromboplastin time (aPTT). For each outcome, patients were excluded only from that specific outcome analysis if the complication was documented prior to the index date. Patients were not excluded from the overall cohort or from other outcome analyses. Laboratory values analyzed had to be collected after index surgery date. This ensured that all results reflect new, incident postoperative events.

### Statistical analysis

All analyses were conducted on the matched cohorts. Propensity score matching (PSM) was performed using 1:1 greedy nearest-neighbor matching with a caliper of 0.10 pooled standard deviations. Matching variables included demographics (age, sex, race), metabolic and medical comorbidities (BMI, HbA1c, osteoporosis, atherosclerotic cardiovascular disease, chronic kidney disease, liver disease, COPD, peripheral arterial disease), polysubstance use disorders (alcohol-, opioid-, and other psychoactive substance-related disorders), polytrauma indicators, preoperative antibiotic administration (identified using CPT-coded perioperative care encounters and medication administration entries documented before operative start time), fracture location, fracture type (open vs. closed), and type of fixation procedure. To further reduce confounding related to surgical approach, all CPT fixation codes used to define the cohort were included as covariates (complete list in Table S1). Concomitant lower extremity injuries were also included as covariates to balance trauma severity and the risk of immobilization-related complications such as VTE. Standardized mean differences (SMD) < 0.10 were used to confirm covariate balance. For binary outcomes, we calculated absolute risk differences, risk ratios (RR), odds ratios (OR), and 95% confidence intervals (CIs). Time-to-event analyses were performed using Kaplan–Meier curves with log-rank testing and Cox proportional hazards models. Continuous outcomes (PT and aPTT) were compared using independent samples t-tests assuming unequal variances. A P value of less than 0.05 was considered statistically significant. To address multiple comparisons across the 21 outcomes tested, Benjamini-Hochberg false discovery rate (FDR) correction was applied. E-values were calculated for significant findings to assess robustness to unmeasured confounding.

## Results

### Sensitivity analysis for cannabis use

A sensitivity analysis was performed to assess the sensitivity of cannabis use in the TriNetX database. According to the National Centers of Drug Use and Health (NSDUH), approximately 6.8% of Americans age 12 or older report having a cannabis use disorder [24]. Applying this prevalence to the over 150 million patients in the TriNetX Research database network would predict roughly 10.2 million cannabis users. However, only 1,666,438 patients (1.11%) in the database carried an ICD-10-CM diagnosis code for cannabis-related disorders.

### Cohort sizes and matching

Before PSM, there were several expected demographic and clinical differences between exposure groups. Cannabis-only users were generally younger and more often male compared with non-users, and nicotine-only users demonstrated a higher comorbidity burden, including greater prevalence of COPD, peripheral arterial disease, and polysubstance use disorders. Differences were also observed in fracture distribution and initial procedural codes. These baseline imbalances are detailed in Table 1 and additional covariates with proper matching can be found in Table S2. After propensity score matching, covariate balance improved substantially, with all SMD < 0.10 for the cannabis-only and concurrent use comparisons, indicating successful matching. In nicotine-only users, age remained slightly above threshold (SMD = 0.14).

**Table 1:** Pre and post matching characteristics of Cannabis-Only, Nicotine-Only, and concurrent users

	Pre-Match: Cannabis-Only Users					Pre-Match: Nicotine-Only Users					Pre-Match: Concurrent Users				
	Cannabis-Only Users (*N* = 930)		Non-Users (*N* = 115,237)		SMD	Nicotine-Only Users (*N* = 14,485)		Non-Users (*N* = 115,237)		SMD	Concurrent Users (*N* = 1,629)		Cannabis-Only Users (*N* = 930)		SMD
	*N*, Mean	%, SD	*N*, Mean	%, SD		*N*, Mean	%, SD	*N*, Mean	%, SD		*N*, Mean	%, SD	*N*, Mean	%, SD	
*Age*,* mean (SD)*	35.33	15.753	45.41	24.21	0.52	47.31	16.28	45.77	24.06	0.08	37.98	13.96	35.27	15.77	0.18
*Male*,* %*	580	71.25%	50,280	43.63%	0.52	10,161	70.15%	91,307	72.59%	0.19	1067	65.50%	625	67.20%	0.04
*Race (White)*	510	62.65%	86,556	75.11%	0.32	7514	51.87%	53,136	42.24%	0.05	899	55.19%	536	57.63%	0.05
*BMI*,* mean (SD)*	26.56	6.305	27.22	6.88	0.12	27.42	6.65	27.29	6.87	0.02	26.27	6.00	26.52	6.20	0.04
18–25 kg/m2	357	43.86%	32,091	27.85%	0.33	5470	37.76%	34,545	27.46%	0.22	793	48.68%	400	43.01%	0.11
25–30 kg/m2	269	33.05%	28,974	25.14%	0.18	5214	36.00%	31,559	25.09%	0.24	643	39.47%	308	33.12%	0.13
30–35 kg/m2	158	19.41%	18,106	15.71%	0.11	3139	21.67%	19,746	15.70%	0.15	355	21.79%	185	19.89%	0.05
35–40 kg/m2	87	10.69%	9166	7.95%	0.08	1556	10.74%	9968	7.92%	0.10	149	9.15%	94	10.11%	0.03
40–60 kg/m2	35	4.30%	5220	4.53%	0.02	868	5.99%	5706	4.54%	0.07	83	5.10%	39	4.19%	0.04
*HbA1c*,* mean (SD)*	5.78	1.33	6.01	1.35	0.17	6.13	1.75	6.10	1.61	0.02	5.93	1.83	5.83	1.45	0.06
4–5.7.7%	100	12.29%	9925	8.61%	0.12	1834	12.66%	11,563	9.19%	0.11	272	16.70%	119	12.80%	0.11
5.7–6.5%	32	3.93%	7833	6.80%	0.13	1258	8.69%	8746	6.95%	0.06	116	7.12%	38	4.09%	0.13
6.5–8.5%	15	1.84%	3728	3.24%	0.10	590	4.07%	4176	3.32%	0.04	44	2.70%	16	1.72%	0.07
8–10%	11	1.35%	1973	1.71%	0.03	381	2.63%	2233	1.78%	0.06	39	2.39%	13	1.40%	0.07
*Comorbidities*															
Hyperlipidemia	85	10.44%	18,864	16.37%	0.17	3043	21.01%	20,544	16.33%	0.12	210	12.89%	97	10.43%	0.08
Cerebrovascular diseases	51	6.27%	5682	4.93%	0.08	1377	9.51%	6488	5.16%	0.17	158	9.70%	65	6.99%	0.10
Diabetes mellitus	58	7.13%	9643	8.37%	0.05	1731	11.95%	10,680	8.49%	0.11	157	9.64%	66	7.10%	0.09
CAD	34	4.18%	5538	4.81%	0.03	1258	8.69%	6108	4.86%	0.15	109	6.69%	40	4.30%	0.11
Other diseases of liver	40	4.91%	3804	3.30%	0.06	984	6.79%	4283	3.41%	0.15	124	7.61%	43	4.62%	0.12
CKD	30	3.69%	4423	3.84%	0.01	672	4.64%	4816	3.83%	0.04	64	3.93%	34	3.66%	0.01
Osteoporosis	10	1.23%	1312	1.14%	0.21	942	6.50%	9738	7.74%	0.05	38	2.33%	28	3.01%	0.04
COPD	22	2.70%	2782	2.41%	0.03	2208	15.24%	3161	2.51%	0.46	172	10.56%	28	3.01%	0.30
PAD	19	2.33%	2118	1.84%	0.02	596	4.12%	2320	1.84%	0.13	44	2.70%	20	2.15%	0.04
Fracture of lower leg	128	15.73%	6632	5.76%	0.34	1806	12.47%	7363	5.85%	0.07	304	18.66%	151	16.24%	0.06

	Post-Match: Cannabis-Only Users					Post-Match: Nicotine-Only Users					Post-Match: Concurrent Users				
	Cannabis-Only Users (N = 801)		Non-Users (N = 801)		**SMD**	Nicotine-Only Users (N = 14, **310)**		Non-Users (N = 14,**310)**		**SMD**	Concurrent Users (N = 901)		Cannabis-Only Users (N = 901)		**SMD**
	**N**,** Mean**	**%**,** SD**	**N**,** Mean**	**%**,** SD**		**N**,** Mean**	**%**,** SD**	**N**,** Mean**	**%**,** SD**		**N**,** Mean**	**%**,** SD**	**N**,** Mean**	**%**,** SD**	
*Age*,* mean (SD)*	35.46	15.82	36.92	19.35	0.08	47.26	16.29	49.91	20.41	0.14	35.01	13.17	35.03	15.54	0.00
*Male*,* %*	569	71.04%	567	70.79%	0.06	10,037	70.14%	10,170	71.07%	0.02	625	69.37%	617	68.48%	0.02
*Race (White)*	501	62.55%	501	62.55%	0.02	7414	51.81%	7263	50.76%	0.02	517	57.38%	518	57.49%	0.01
*BMI*,* mean (SD)*	26.56	6.30	26.92	5.76	0.05	27.43	6.66	27.85	6.42	0.06	26.11	5.99	26.40	6.14	0.05
18–25 kg/m2	351	43.82%	346	43.20%	0.05	5360	37.46%	5451	38.09%	0.01	403	44.73%	393	43.62%	0.02
25–30 kg/m2	267	33.33%	297	37.08%	0.08	5110	35.71%	5549	38.78%	0.06	301	33.41%	303	33.63%	0.01
30–35 kg/m2	154	19.23%	170	21.22%	0.02	3079	21.52%	3474	24.28%	0.07	174	19.31%	173	19.20%	0.00
35–40 kg/m2	84	10.49%	89	11.11%	0.04	1537	10.74%	1751	12.24%	0.05	78	8.66%	84	9.32%	0.02
40–60 kg/m2	35	4.37%	31	3.87%	0.03	855	5.98%	958	6.70%	0.03	32	3.55%	39	4.33%	0.04
*HbA1c*,* mean (SD)*	5.78	1.34	5.75	1.13	0.01	6.12	1.75	6.13	1.69	0.01	5.96	1.71	5.80	1.45	0.09
4–5.7.7%	98	12.24%	92	11.49%	0.02	1795	12.54%	1874	13.10%	0.02	102	11.32%	113	12.54%	0.03
5.7–6.5%	32	4.00%	29	3.62%	0.01	1237	8.64%	1321	9.23%	0.02	36	4.00%	35	3.89%	0.01
6.5–8.5%	15	1.87%	14	1.75%	0.02	581	4.06%	646	4.51%	0.02	15	1.67%	13	1.44%	0.02
8–10%	11	1.37%	12	1.50%	0.03	374	2.61%	417	2.91%	0.02	14	1.55%	11	1.22%	0.03
*Comorbidities*															
Hyperlipidemia	85	10.61%	103	12.86%	0.07	2983	20.85%	3164	22.11%	0.03	90	9.99%	86	9.55%	0.01
Cerebrovascular diseases	51	6.37%	44	5.49%	0.03	1343	9.39%	1397	9.76%	0.01	58	6.44%	65	7.21%	0.03
Diabetes mellitus	58	7.24%	62	7.74%	0.03	1694	11.84%	1851	12.94%	0.03	59	6.55%	63	6.99%	0.02
CAD	34	4.25%	38	4.74%	0.02	1225	8.56%	1320	9.22%	0.02	38	4.22%	38	4.22%	0.01
Other diseases of liver	40	4.99%	34	4.25%	0.03	958	6.70%	938	6.56%	0.01	35	3.89%	40	4.44%	0.03
CKD	30	3.75%	29	3.62%	0.02	663	4.63%	709	4.96%	0.02	33	3.66%	31	3.44%	0.01
Osteoporosis	23	2.87%	25	3.12%	0.03	934	6.53%	1060	7.41%	0.03	19	2.11%	18	2.00%	0.01
COPD	22	2.75%	22	2.75%	0.04	2095	14.64%	1998	13.96%	0.02	32	3.55%	28	3.11%	0.02
PAD	19	2.37%	23	2.87%	0.01	578	4.04%	567	3.96%	0.00	18	2.00%	19	2.11%	0.02
Fracture of lower leg	126	15.73%	138	17.23%	0.03	1750	12.23%	1787	12.49%	0.01	144	15.98%	145	16.09%	0.02

Following propensity score matching, cannabis-only users vs. non-users comparison included 801 patients per group; the nicotine-only users vs. non-user controls comparison included 14,310 patients per group; and the concurrent users vs. cannabis-only users comparison included 901 patients per group. Results of pre and post matching can be found in Table 1. A full summary of fracture types after matching is provided in Table S2. Across comparisons of cannabis-only, nicotine-only, and concurrent users, baseline characteristics including age, sex, race, fracture type, and comorbidities were balanced with SMD < 0.10, confirming successful covariate matching. However, covariate balance was not fully achieved in the nicotine-only user cohort, with some select variables exhibiting SMD > 0.10, indicating residual imbalance despite matching.

### Cannabis-only users

**Fig. 2:**
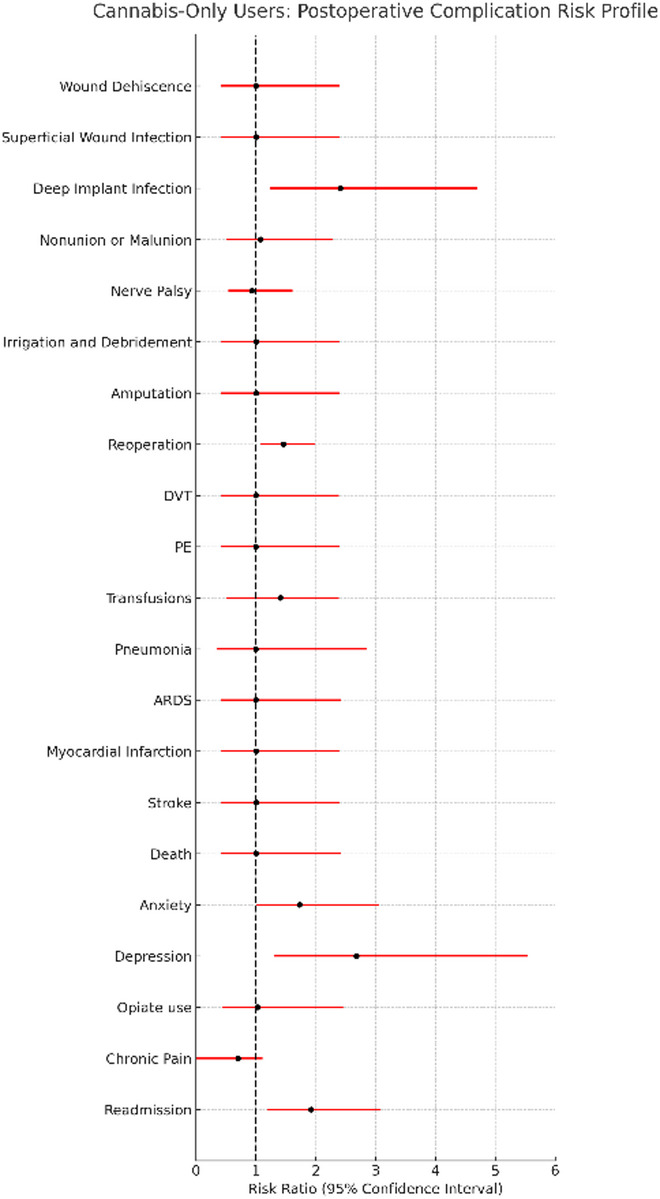
Cannabis-Only Users: Postoperative Complication Risk Profile Risk ratio profile comparing cannabis-only users to matched non-user controls following surgical fixation of upper extremity fractures. Each risk ratio represents the relative likelihood of postoperative complications among cannabis-only users versus non-users after propensity score matching

Cannabis-only users demonstrated higher rates of several postoperative complications compared with matched non-users, including deep implant infection, reoperation, readmission, and new-onset depression and anxiety. Specific values for these outcomes are reported in Table 2, and comparative risk ratio profiles are shown in Fig. 2.

**Table 2:** Outcomes of upper extremity surgical fixation in Cannabis-Only, Nicotine-Only, and concurrent users

Outcome	Cannabis-Only Users vs. Non-Users (*N* = 801)						Nicotine-Only Users vs. Non-Users (*N* = 14,310)						Concurrent Users vs. Cannabis-Only Users (*N* = 901)					
	Cannabis *n* (%)	Non-User *n* (%)	RD (%)	95% CI	RR	*P*	Nicotine *n* (%)	Non-User *n* (%)	RD (%)	95% CI	RR	*P*	Concurrent *n* (%)	Cannabis *n* (%)	RD (%)	95% CI	RR	*P*
Wound Dehiscence	23 (2.9%)	13 (1.6%)	0.42	(−0.07,0.90)	1.77	0.093	459 (3.2%)	262 (1.8%)	0.54	(0.39,0.68)	1.75	**< 0.001**	28 (3.1%)	21 (2.3%)	0.30	(−0.28,0.89)	1.34	0.312
Superficial Wound Infection	89 (11.1%)	79 (9.9%)	0.36	(−0.62,1.64)	1.14	0.376	1844 (12.9%)	1319 (9.2%)	1.75	(1.41,2.08)	1.44	**< 0.001**	137 (15.2%)	85 (9.4%)	2.82	(1.44,4.21)	1.70	**< 0.001**
Deep Implant Infection	63 (7.9%)	28 (3.5%)	1.47	(0.70,2.24)	2.26	**< 0.001**	1172 (8.2%)	769 (5.4%)	1.11	(0.88,1.35)	1.53	**< 0.001**	97 (10.8%)	56 (6.2%)	1.80	(0.76,2.83)	1.74	**< 0.001**
Nosocomial Infection	≤ 10*	≤ 10*	0	(−0.36,0.36)	1.00	0.994	123 (0.9%)	79 (0.6%)	0.12	(0.04,0.20)	1.56	**0.002**	≤ 10*	≤ 10*	0	(−0.38,0.38)	1.00	0.999
Nonunion/Malunion	158 (19.7%)	109 (13.6%)	2.14	(0.81,3.46)	1.46	**0.002**	1296 (9.1%)	916 (6.4%)	1.06	(0.81,1.31)	1.42	**< 0.001**	107 (11.9%)	82 (9.1%)	1.13	(−0.02,2.29)	1.32	0.054
Nerve Palsy	28 (3.5%)	13 (1.6%)	0.64	(0.11,1.18)	2.17	**0.017**	362 (2.5%)	281 (2.0%)	0.24	(0.10,0.38)	1.30	**< 0.001**	23 (2.6%)	27 (3.0%)	−0.16	(−0.78,0.45)	0.86	0.604
I&D	18 (2.2%)	≤ 10*	0.34	(−0.10,0.77)	1.81	0.128	250 (1.7%)	190 (1.3%)	0.17	(0.06,0.29)	1.33	**0.003**	25 (2.8%)	19 (2.1%)	0.28	(−0.29,0.85)	1.34	0.332
Amputation	≤ 10*	≤ 10*	0	(−0.36,0.36)	1.00	1.000	17 (0.1%)	≤ 10*	0.02	(−0.01,0.05)	1.70	0.177	≤ 10*	0 (0%)	0.43	(0.16,0.69)	—	**0.002**
Reoperation	356 (44.4%)	274 (34.2%)	3.39	(1.49,5.28)	1.30	**< 0.001**	4824 (33.7%)	3903 (27.3%)	2.49	(2.03,2.96)	1.24	**< 0.001**	432 (47.9%)	335 (37.2%)	4.15	(2.03,6.27)	1.29	**< 0.001**
DVT	≤ 10*	≤ 10*	0	(−0.36,0.36)	1.00	0.999	119 (0.8%)	100 (0.7%)	0.05	(−0.03,0.13)	1.19	0.200	≤ 10*	≤ 10*	0	(−0.38,0.38)	1.00	0.999
PE	40 (5.0%)	33 (4.1%)	0.29	(−0.40,0.98)	1.21	0.409	699 (4.9%)	647 (4.5%)	0.14	(−0.05,0.33)	1.08	0.153	39 (4.3%)	39 (4.3%)	0	(−0.73,0.73)	1.00	1.000
Transfusions	77 (9.6%)	46 (5.7%)	1.39	(0.46,2.31)	1.71	**0.003**	1264 (8.8%)	1009 (7.1%)	0.75	(0.49,1.01)	1.27	**< 0.001**	94 (10.4%)	69 (7.7%)	1.15	(0.04,2.26)	1.37	**0.042**
Pneumonia	22 (2.7%)	26 (3.2%)	−0.18	(−0.76,0.41)	0.84	0.556	897 (6.3%)	727 (5.1%)	0.51	(0.28,0.74)	1.24	**< 0.001**	36 (4.0%)	20 (2.2%)	0.75	(0.09,1.41)	1.84	**0.025**
Respiratory Failure	22 (2.7%)	24 (3.0%)	−0.07	(−0.66,0.52)	0.93	0.814	897 (6.3%)	855 (6.0%)	0.17	(−0.07,0.42)	1.07	0.171	38 (4.2%)	20 (2.2%)	0.88	(0.18,1.58)	1.94	**0.014**
ARDS	≤ 10*	≤ 10*	0	(−0.36,0.36)	1.00	1.000	179 (1.3%)	147 (1.0%)	0.09	(−0.01,0.18)	1.22	0.076	≤ 10*	≤ 10*	0	(−0.37,0.37)	1.00	1.000
MI	13 (1.6%)	≤ 10*	0.13	(−0.26,0.52)	1.31	0.516	392 (2.7%)	339 (2.4%)	0.15	(0.01,0.30)	1.16	**0.042**	≤ 10*	12 (1.3%)	−0.09	(−0.49,0.32)	0.84	0.676
AKI	30 (3.7%)	28 (3.5%)	0.12	(−0.54,0.79)	1.10	0.713	818 (5.7%)	846 (5.9%)	−0.06	(−0.30,0.19)	0.98	0.648	42 (4.7%)	28 (3.1%)	0.72	(−0.06,1.49)	1.55	0.069
Stroke	13 (1.6%)	≤ 10*	0.13	(−0.27,0.52)	1.31	0.522	262 (1.8%)	274 (1.9%)	−0.03	(−0.16,0.10)	0.96	0.634	≤ 10*	12 (1.3%)	−0.09	(−0.49,0.31)	0.83	0.671
Death	44 (5.5%)	46 (5.7%)	−0.08	(−0.84,0.68)	0.96	0.833	1828 (12.8%)	1538 (10.7%)	0.80	(0.49,1.10)	1.19	**< 0.001**	41 (4.6%)	42 (4.7%)	−0.04	(−0.80,0.72)	0.98	0.912
Anxiety	88 (11.0%)	49 (6.1%)	2.62	(1.44,3.81)	2.15	**< 0.001**	1351 (9.4%)	1055 (7.4%)	1.47	(1.14,1.80)	1.42	**< 0.001**	109 (12.1%)	84 (9.3%)	1.89	(0.30,3.47)	1.39	**0.019**
Depression	66 (8.2%)	35 (4.4%)	1.52	(0.65,2.40)	2.01	**< 0.001**	894 (6.2%)	627 (4.4%)	0.90	(0.67,1.13)	1.49	**< 0.001**	72 (8.0%)	63 (7.0%)	0.54	(−0.55,1.63)	1.18	0.329
Opioid Use	18 (2.2%)	≤ 10*	0.35	(−0.09,0.78)	1.83	0.118	306 (2.1%)	91 (0.6%)	0.60	(0.49,0.71)	3.43	**< 0.001**	37 (4.1%)	17 (1.9%)	0.97	(0.32,1.62)	2.31	**0.003**
Chronic Pain	103 (12.9%)	95 (11.9%)	0.63	(−0.61,1.87)	1.15	0.317	2086 (14.6%)	1451 (10.1%)	2.39	(2.02,2.75)	1.53	**< 0.001**	149 (16.5%)	100 (11.1%)	2.68	(1.18,4.17)	1.55	**< 0.001**
Readmission	183 (22.8%)	141 (17.6%)	1.74	(0.33,3.14)	1.30	**0.016**	2260 (15.8%)	1502 (10.5%)	2.05	(1.73,2.37)	1.51	**< 0.001**	281 (31.2%)	172 (19.1%)	4.66	(2.97,6.35)	1.63	**< 0.001**

Bold value represents statistical significance for an outcome event. The asterisk indicates that there are less than 10 events for an outcome which is censored by TriNetX for privacy protection of patients

**Fig. 3:**
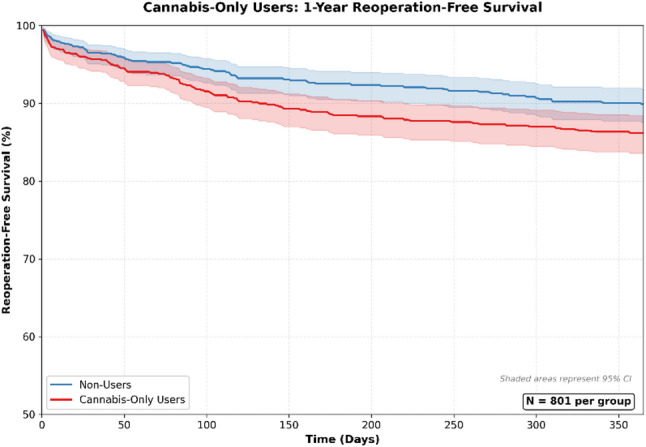
Cannabis-Only Users: 1 Year Reoperation Survival Curve

Deep implant infection was significantly more frequent in cannabis-only users (risk difference: 1.85%; 95% CI: 0.50–3.21%). Elevated risks were also observed for reoperation (risk difference: 3.62%; 95% CI: 0.73–6.51%) and readmission (risk difference: 2.87%; 95% CI: 0.83–4.91%). Survival curves for reoperation for cannabis-only users can be found in Fig. 3. Rates of depression (risk difference: 2.21%; 95% CI: 0.68–3.80%) and anxiety (risk difference: 2.03%; 95% CI: 0.03–4.02%) were also higher; these represent new postoperative diagnoses, as individuals with prior documentation of these conditions were excluded from the respective analyses.

No significant difference were identified for wound complications (dehiscence, superficial infection), nonunion or malunion, irrigation and debridement, amputation, nerve palsy, pneumonia, ARDS, blood transfusion, DVT, PE, MI, stroke, mortality, chronic pain, opiate use, or coagulation parameters (PT, aPTT) (Table 3).

**Table 3:** Comparative analysis of coagulation profiles among Cannabis-Only, Nicotine-Only, and concurrent users

Outcome	Comparison	Mean (Group 1)	SD (Group 1)	Mean (Group 2)	SD (Group 2)	t	df	*P*-value
Partial Thromboplastin Time (PTT)	Cannabis-Only Users vs. Non-Users	33.169	10.88	34.193	12.733	−0.525	153	0.6001
	Nicotine-Only Users vs. Non-Users	33.343	11.392	33.914	13.445	−1.304	3337	0.1924
	Concurrent Users vs. Cannabis Users	33.511	11.362	34.183	13.615	−0.398	225	0.6908
Prothrombin Time (PT)	Cannabis-Only Users vs. Non-Users	13.548	3.157	13.526	2.955	0.049	205	0.9612
	Nicotine-Only Users vs. Non-Users	13.736	5.105	14.165	5.45	−2.567	4198	0.0103
	Concurrent Users vs. Cannabis Users	13.818	4.494	13.391	2.547	1.058	313	0.2909

After Benjamini-Hochberg FDR correction across 21 outcomes, only readmission remained statistically significant (q = 0.006). Deep implant infection and depression showed borderline significance (q = 0.051). Reoperation and anxiety remained nominally significant (*p* < 0.05) but did not retain significance after correction. E-values indicated moderate to strong robustness to unmeasured confounding, with deep implant infection demonstrating the highest E-value (4.31). Number Needed to Harm (NNH) values were 35 for readmission, 28 for reoperation, 45 for depression, 49 for anxiety, and 54 for deep implant infection.

### Nicotine-only users

**Fig. 4:**
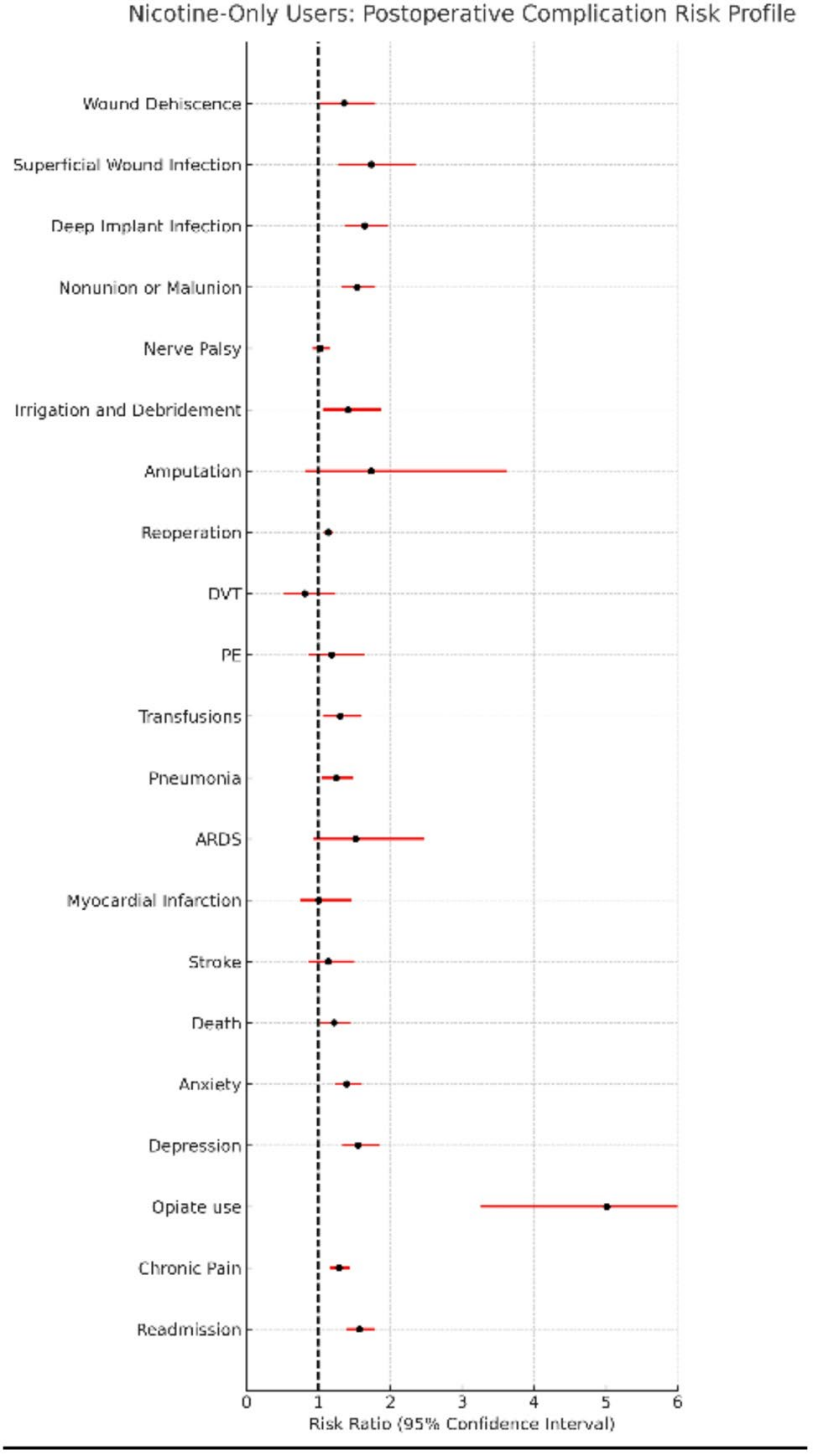
Nicotine-Only Users: Postoperative Complication Risk Profile Risk ratio profile comparing nicotine-only users to matched non-user controls following surgical fixation of upper extremity fractures. Each risk ratio represents the relative likelihood of postoperative complications among nicotine-only users versus non-users after propensity score matching

Nicotine-only users demonstrated a broader and more pronounced pattern of postoperative complications compared with matched non-users, with significantly elevated risks across multiple surgical, medical, and psychological outcomes. Detailed values are provided in Table 2, and risk ratio profiles are displayed in Fig. 4.

**Fig. 5:**
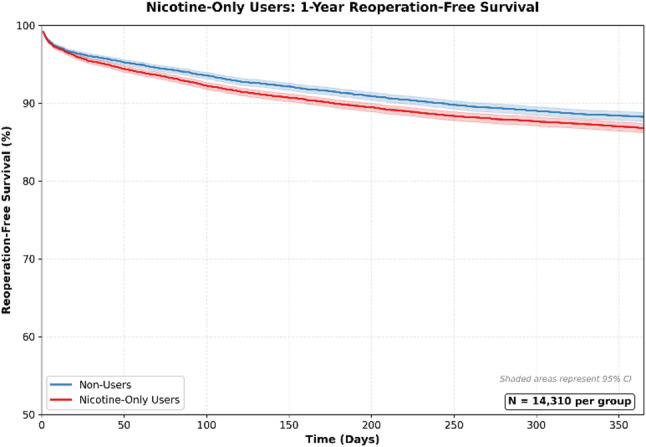
Nicotine-Only Users: 1 Year Reoperation Survival Curve

Significant increases were observed in deep implant infection (risk difference: 0.84%; 95% CI: 0.54–1.36%), superficial surgical site infection (risk difference: 0.34%; 95% CI: 0.15–0.52%), wound dehiscence (risk difference: 0.21%; 95% CI: 0.02–0.36%), and nonunion or malunion (risk difference: 1.01%; 95% CI: 0.65–1.36%). Reoperations (risk difference: 1.41%; 95% CI: 0.68–2.13%), irrigation and debridement (risk difference: 0.23%; 95% CI: 0.04–0.42%), and readmissions (risk difference: 1.65%; 95% CI: 1.21–2.09%) were all significantly more frequently in nicotine-only users. Survival curves for reoperation for nicotine-only users can be found in Fig. 5.

Medical complications were also elevated, with higher rates of mortality (risk difference: 0.34%; 95% CI: 0.05–0.63%) and pneumonia (risk difference: 0.40%; 95% CI: 0.08–0.72%). Psychosocial complications were more common as well, including new postoperative diagnoses of depression (risk difference: 1.01%; 95% CI: 0.64–1.37%), anxiety (risk difference: 1.27%; 95% CI: 0.90–1.88%), opiate use disorder (risk difference: 0.71%; 95% CI: 0.54–0.88%), and chronic pain (risk difference: 1.25%; 95% CI: 0.70–1.80%). As with the cannabis-only user analysis, individuals with prior psychological diagnoses were excluded from these analyses to ensure assessment of new post-operative events.

No significant differences were observed in nerved palsy, amputation, blood transfusion, DVT, PE, ARDS, MI, stroke, or aPTT (Tables 2 and 3). PT values differed statistically (mean 13.70 vs. 14.24; *p* = 0.0015), but were not clinically significant (Reference Range 11.0–13.5 s).

After Benjamini–Hochberg FDR correction, 12 outcomes remained statistically significant, including deep implant infection, nonunion/malunion, reoperation, anxiety, depression, opioid use disorder, chronic pain, readmission, superficial wound infection, pneumonia, irrigation and debridement, and death, with wound dehiscence borderline significant. E-values ranged from 1.53 (reoperation) to 9.52 (opioid use disorder), demonstrating moderate-to-strong robustness to unmeasured confounding. Number Needed to Harm (NNH) estimates ranged from 61 (readmission) to 485 (wound dehiscence).

### Concurrent users

**Fig. 6:**
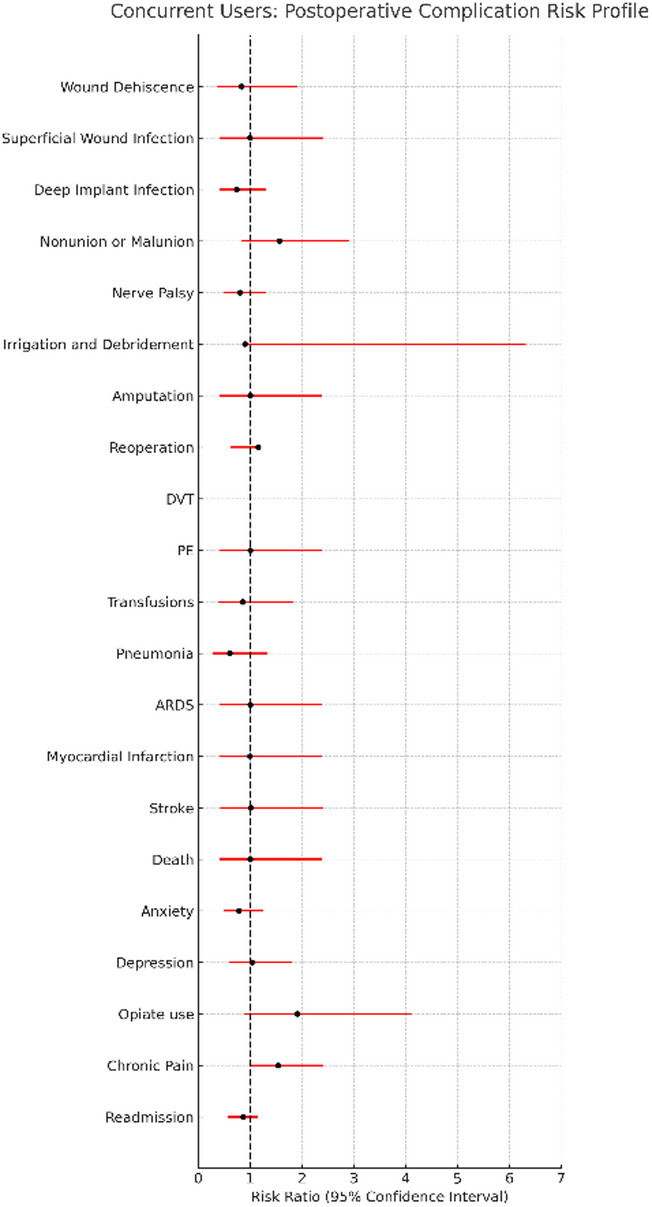
Concurrent Users: Postoperative Complication Risk Profile Risk ratio profile comparing concurrent cannabis-and-nicotine users to matched cannabis-only users following surgical fixation of upper extremity fractures. Each risk ratio represents the relative likelihood of postoperative complications among concurrent users versus cannabis-only users after propensity score matching

Concurrent users did not demonstrate significantly higher postoperative complication rates compared with matched cannabis-only users, and no outcome showed statistically significant additive or synergistic risk. Full numerical results are shown in Table 2, with corresponding risk ratio profiles in Fig. 6.

**Fig. 7:**
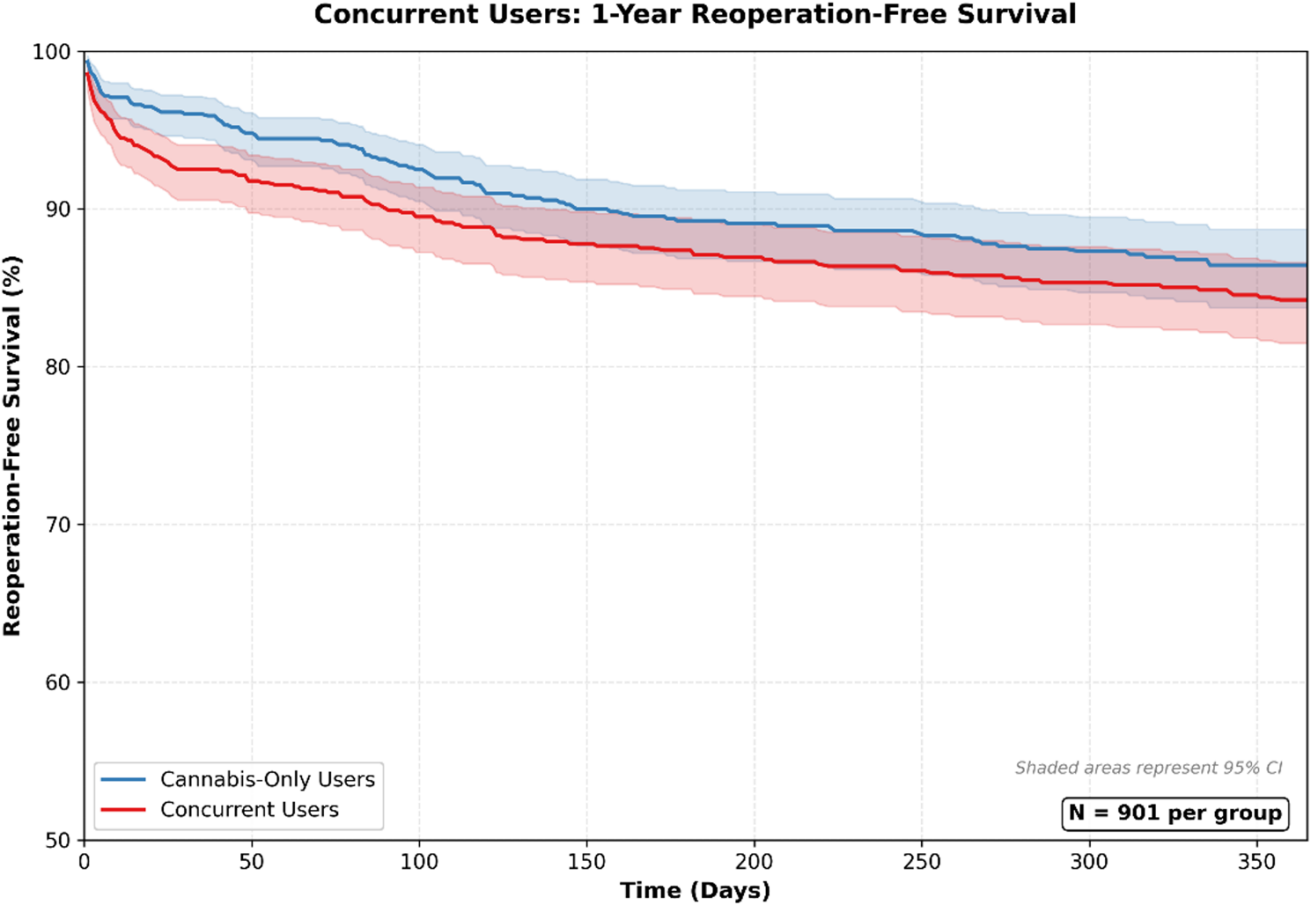
Concurrent Users: 1 Year Reoperation Survival Curve

Across surgical, medical, and psychosocial outcomes, rates in concurrent users were generally comparable to those in cannabis-only users. Although the risk of reoperation was numerically higher in concurrent users (risk difference: 2.66%, *p* = 0.09), the difference did not reach statistical significance. Survival curves for reoperations in concurrent users can be found in Fig. 7. No significant differences were observed for deep implant infection, superficial infection, wound dehiscence, nonunion or malunion, irrigation and debridement, readmission, chronic pain, anxiety, depression, opioid use disorder, or mortality. Coagulation parameters (PT, aPTT) were also similar between groups (Table 3).

After Benjamini–Hochberg FDR correction, no outcomes met criteria for statistical significance. This null finding reflects both the limited sample size and the small risk differences observed. Post-hoc power analysis demonstrated that with approximately 900 patients per group, the comparison had 80% power to detect risk differences of roughly 3% for outcomes with 5% baseline incidence, suggesting that smaller effects may have been missed.

## Discussion

In this large multi-institutional cohort, cannabis-only and nicotine-only use were each independently associated with increased postoperative complications following upper extremity fracture fixation. Both cannabis and nicotine use were independently associated with significantly increased risks of surgical and medical complications including implant-related infection, readmission, reoperation, and mood disorders. Nicotine use was additionally associated with elevated rates of non-union, malunion, wound dehiscence, superficial wound infection, need for irrigation and debridement, pneumonia, mortality, and chronic pain. Notably, there were no significant additive effects observed among concurrent users. These findings are particularly relevant given rising rates of cannabis use especially amongst young men who are disproportionately affected by orthopedic trauma [25, 26]. 

The robustness of our findings to unmeasured confounding was assessed using E-values. The E-value of 4.31 for deep implant infection indicates that an unmeasured confounder would need a risk ratio of at least 4.31 with both cannabis use and infection to fully explain away the observed association. Given the comprehensive propensity score matching on demographics, comorbidities, fracture characteristics, and polysubstance use, such a strong unmeasured confounder seems unlikely, supporting a potential causal relationship. However, the borderline significance of several outcomes after FDR correction warrants cautious interpretation.

Cannabis-only users exhibited a higher rate of deep implant infection compared to non-users. This finding is consistent with prior studies reporting increased superficial and deep infections among cannabis users [8, 10, 11, 27]. Cannabinoids may impair key antimicrobial immune function including phagocytosis, oxidative burst, antigen presentation, and adaptive immune cell recruitment, which may meaningfully weaken host defense in the peri-implant environment [4–6, 28]. These immunomodulatory effects can compromise fracture healing and reduce resistance to biofilm-forming pathogens [7]. Further work is needed to determine whether cannabis users are more susceptible to specific pathogen profiles and to evaluate whether targeted strategies such as extended systemic prophylaxis or local antimicrobial delivery can mitigate this risk [29–32]. 

Furthermore, cannabis-only users demonstrated significantly higher reoperation rates reflecting the need to address the increase in deep implant infection rates. These findings are corroborated by Turan et al., who demonstrated increased rates of both reoperation and periprosthetic joint infection (PJI) in cannabis users following total joint arthroplasty procedures [33]. Despite reports of delayed bone healing and prolonged time to union in prior literature, we did not detect statistically significant differences in non-union or malunion in this retrospective cohort [8, 34, 35]. Further prospective and basic science research are warranted to further characterize the downstream effects of cannabinoids on bone healing and immunity.

Psychosocial complications were also seen more frequently with cannabis users, demonstrating increased rates of readmission, anxiety, and depression as compared to non-users. Our findings linking cannabis use to mood disorders is supported by existing literature that highlights prolonged cannabis use to increased odds of developing mood and anxiety disorders [36, 37]. This relationship is further strengthened by observations by Langlois et al., in which authors explore a “bi-directional” relationship between cannabis use and depression, where the use of cannabis increases the rates of depression and subsequent cannabis use in a positive feedback loop [38]. 

In our matched cohort, cannabis use was associated with a 4% higher rate of readmission compared to non-users, with a NNH of 35—meaning one additional readmission would be expected for every 35 cannabis users undergoing upper extremity fracture fixation compared to non-users. The elevated readmission rate may reflect surgical complications (infection, reoperation), medical complications, psychosocial factors (the elevated depression and anxiety rates observed in this cohort), or healthcare access barriers. Notably, readmission was the only outcome that definitively survived Benjamini-Hochberg FDR correction. These patients may benefit from enhanced pre-discharge communication, integrated mental health screening, and close outpatient follow-up [39]. Taken together, these psychosocial risks support the need for pre-discharge interventions and integrated mental health screening to support this population.

The nicotine-only users cohort was designed as an internal-control, given the well-established effects of nicotine on respiratory and cardiovascular health and its known association with surgical complications such as infection and nonunion in orthopaedic surgery. As expected, nicotine use was independently associated with increased risks of postoperative medical and psychosocial complications, including higher mortality. These findings likely reflect the documented impact of nicotine on neutrophil function, wound healing, endothelial integrity, and systemic inflammation [40–42]. In addition, nicotine users demonstrated elevated rates of surgical complications including wound dehiscence, nonunion or malunion, and reoperation, associations that have been consistently reported in prior literature [42–44]. 

Concurrent use of cannabis and nicotine was not associated with a significantly greater risk of complications compared to cannabis use alone. However, several important limitations temper interpretation of this null finding. First, the concurrent users comparison (*N* = 901 per group) was underpowered to detect small-to-moderate additive effects; post-hoc power analysis indicated 80% power to detect risk differences of approximately 3% for outcomes with 5% baseline rates. Second, formal interaction testing was not available within the TriNetX platform. Third, the absence of statistically significant additive effects may reflect overlapping biological pathways between cannabis and nicotine, or a true absence of synergistic effects. Future studies with larger concurrent use cohorts and formal interaction testing are needed to clarify this relationship.

Although this study was not designed to test specific perioperative interventions, our findings have several practical implications for clinical care. First, cannabis use should be routinely screened for during preoperative evaluation of patients undergoing upper extremity fracture fixation, as it was independently associated with higher rates of implant related infection, reoperation, readmission, and mood disorders. Second, recognition of cannabis exposure may prompt closer postoperative monitoring for wound complications, infection, mental health symptoms, and return to the emergency department or hospital. Third, surgeons may consider counseling patients regarding these potential risks as part of shared decision making and discharge planning. These practice considerations, while not directly tested in this cohort, are supported by the observed associations and can help guide risk stratification while future work evaluates targeted interventions. These recommendations have not been prospectively tested, and prospective studies are needed to determine whether they reduce postoperative risk.

### Limitations

This study has several limitations. First, reliance on ICD-10 coding may introduce misclassification bias due to variability in documentation and coding practices across healthcare organizations. Substance use may be underreported due to stigma, lack of routine screening, or patient non-disclosure, which could bias results toward the null [45, 46]. Additionally, the TriNetX database does not capture granular details regarding the frequency, quantity, timing, or route of cannabis or nicotine use, all of which may influence complication risk. Cannabis diagnoses are known to be substantially under-ascertained in this platform, with an estimated sixfold relative to national prevalence, likely related to inconsistent screening, documentation stigma, or prioritization of primary diagnoses during. As a result, the true magnitude of the associations observed in this study may differ if cannabis exposure were more comprehensively captured and may in fact be greater than reported.

While efforts were made to control for polysubstance use by adjusting for alcohol, opioids, and other psychoactive substances, unmeasured confounding may remain. Socioeconomic status and mental health, both of which may mediate the relationship between substance use and postoperative outcomes, could not be assessed due to limitations in available data and coding.

Injury Severity Scores (ISS) were also unavailable. To partially address this limitation, we adjusted for concurrent lower extremity injuries and used umbrella codes for nonspecific multiple-injury codes to account for polytrauma, although unmeasured injury patterns and trauma severity may still contribute to observed complications. Similarly, the Charlson Comorbidity Index (CCI) was not directly available, though its component diagnoses were incorporated into propensity score matching.

Timing-related variables were also limited. Time to antibiotic administration in open injuries could not be reliably determined within the platform. While we accounted for whether preoperative antibiotics were administered, we were unable to assess the use of topical antimicrobial formulations or prolonged antibiotic therapy among patients who developed infections after the index injury.

Although short-interval postoperative windows (such as 30-day or 60-day outcomes) are clinically meaningful, these analyses were not feasible because narrowing the follow-up window resulted in event counts below the TriNetX minimum cell-size threshold (< 10 patients), triggering automatic suppression. Consequently, all outcomes were analyzed over a standardized 1-year postoperative period to ensure sufficient event counts and comparability across exposure groups.

The TriNetX platform does not report missing data rates by variable, and multiple imputation methods were not available. Doubly-robust estimation techniques (e.g., augmented inverse probability weighting) were similarly not supported. Additionally, formal testing of the proportional hazards assumption (e.g., Schoenfeld residuals) could not be performed; Kaplan–Meier curves were visually assessed and showed consistent separation without crossing, providing indirect support for this assumption. The platform also anonymizes contributing healthcare organizations, preventing adjustment for site-level clustering or temporal trends based on calendar year.

Reoperation events could not be stratified by the type of surgical procedure or by injury location because subsequent procedures are reported in TriNetX only as aggregated procedural categories without CPT-level detail linking the event to a specific anatomical site. This limitation prevented a more granular characterization of reoperation subtype or etiology. Additionally, only all-cause readmission could be measured; the platform does not support categorization of readmission by surgical, medical, or psychosocial cause. This limitation restricts interpretation of underlying readmission drivers, which may reflect surgical complications, medical events, psychosocial factors, or access-to-care barriers.

We were unable to perform sensitivity analyses using stricter cannabis exposure definitions—such as requiring multiple F12 encounters—or to substratify by fracture type or anatomical location, because these approaches reduced event counts below the TriNetX privacy threshold or prevented successful matching. The platform also does not provide access to clinician notes or toxicology data, limiting our ability to further refine exposure specificity.

Despite these limitations, the use of a large, nationally representative dataset and rigorous propensity score matching substantially strengthens internal validity and supports the generalizability of these findings.

## Conclusion

In this large retrospective database study, both cannabis and nicotine use were independently associated with increased postoperative complications following surgical fixation of upper extremity fractures. Cannabis use was linked to significantly higher rates of implant-related infection, reoperation, readmission, and mood disorders. These findings raise concern for cannabis-mediated impairments in antimicrobial immunity, bone healing, and psychosocial recovery. Nicotine use was associated with an even broader range of adverse outcomes, including wound dehiscence, superficial and deep infection, nonunion or malunion, pneumonia, chronic pain, and increased mortality, findings consistent with its well-established effects on immune and vascular health. Concurrent use of cannabis and nicotine did not demonstrate statistically significant additive effects, though this comparison was limited by sample size and by the absence of formal interaction testing; therefore, the lack of detected synergy should not be interpreted as conclusive evidence against additive biological effects. Taken together, these findings underscore the importance of standardized substance-use screening and targeted perioperative planning for orthopaedic trauma patients. Future prospective and translational studies are needed to define substance-specific microbial patterns, immunologic pathways, and optimal prophylactic strategies, including extended antibiotic coverage and local antimicrobial delivery, to reduce complications and improve outcomes in this growing at-risk population.

## Electronic Supplementary Material

Below is the link to the electronic supplementary material.

Supplementary Material 1

## Data Availability

BMC encourages the sharing of clinical datasets; however, the data used in this study were obtained from the TriNetX Research Network (Cambridge, MA; www.trinetx.com), a federated third-party database containing de-identified electronic health record data. The authors do not have the legal authority to publicly share or deposit the raw patient-level data in a public repository because access is governed by TriNetX licensing agreements and by privacy protections for clinical data.The data generated and analyzed for this study are included in this published article. Researchers who wish to replicate or extend the analyses may obtain access TriNetX directly through its Research Network. Access can be requested from TriNetX, subject to institutional participation and data use agreement. The authors have provided a full list of query parameters, cohort definitions, and analytic criteria to support reproducibility.
