# Supplementary material for: Cannabis and nicotine use are independently associated with adverse surgical, medical, and psychosocial outcomes following upper extremity fracture fixation
Source: J Orthop Surg Res. 2026 Jan 19;21:127. doi: 10.1186/s13018-025-06635-w (PMC12903482; doi:10.1186/s13018-025-06635-w)
Supplement: Supplementary file 1 — Supplementary Material 1 [file 13018_2025_6635_MOESM1_ESM.docx]

**Supplemental Table 1 – Coding Definitions**

| **Category** | **Variable** | **Code(s)** | **Description** |
| --- | --- | --- | --- |
| **Exposure Definitions** | | | |
| Exposure | Cannabis Use Disorder | ICD-10-CM: F12.10, F12.11, F12.12, F12.13, F12.14, F12.15, F12.16, F12.17, F12.18, F12.19, F12.20, F12.21, F12.22, F12.23, F12.24, F12.25, F12.26, F12.27, F12.28, F12.29, F12.90, F12.91, F12.92, F12.93, F12.94, F12.95, F12.96, F12.97, F12.98, F12.99 | Cannabis-related disorders including abuse, dependence, and use |
| Exposure | Nicotine Dependence | ICD-10-CM: F17.200, F17.201, F17.203, F17.208, F17.209, F17.210, F17.211, F17.213, F17.218, F17.219, F17.220, F17.221, F17.223, F17.228, F17.229, F17.290, F17.291, F17.293, F17.298, F17.299 | Nicotine dependence with and without complications |
| **Surgical Outcomes** | | | |
| Outcome | Wound Dehiscence | ICD-10-CM: T81.30XA, T81.31XA, T81.32XA, T81.33XA | Disruption of wound, unspecified, and by body region |
| Outcome | Superficial Surgical Site Infection | ICD-10-CM: T81.41XA | Infection following a procedure, superficial incisional surgical site |
| Outcome | Deep Implant Infection | ICD-10-CM: T84.50XA, T84.51XA, T84.52XA, T84.53XA, T84.54XA, T84.59XA, T84.60XA, T84.61XA, T84.62XA, T84.63XA, T84.64XA, T84.69XA, T84.7XXA | Infection and inflammatory reaction due to internal fixation device |
| Outcome | Nosocomial Infection | ICD-10-CM: A41.9, A49.9, B95-B97 | Healthcare-associated infections |
| Outcome | Nonunion/Malunion | ICD-10-CM: M84.0, M84.1, M84.2; S42.xxx [K, M, N, P], S52.xxx [K, M, N, P], S62.xxx [K, M, N, P] | Nonunion and malunion of fracture; 7th character K=nonunion, M/N=malunion, P=subsequent encounter |
| Outcome | Nerve Palsy | ICD-10-CM: G56.00, G56.01, G56.02, G56.10, G56.11, G56.12, G56.20, G56.21, G56.22, G56.30, G56.31, G56.32, G56.40, G56.41, G56.42, G56.80, G56.81, G56.82, G56.90, G56.91, G56.92 | Mononeuropathies of upper limb including carpal tunnel, ulnar, radial nerve lesions |
| Outcome | Irrigation and Debridement | CPT: 11042, 11043, 11044, 11045, 11046, 11047, 97597, 97598 | Debridement of wound, skin, subcutaneous tissue, muscle, bone |
| Outcome | Amputation | CPT: 24900, 24920, 24930, 24931, 24935, 24940, 25900, 25905, 25907, 25909, 25915, 25920, 25922, 25924, 25927, 26910, 26951, 26952 | Amputation of upper extremity at various levels |
| Outcome | Reoperation | CPT: 20680, 20670, 24160, 24164, 25248, 25250, 25251, 26320 | Removal of implant, deep; removal of internal fixation device |
| **Medical Outcomes** | | | |
| Outcome | Deep Vein Thrombosis (DVT) | ICD-10-CM: I82.40, I82.401, I82.402, I82.403, I82.409, I82.41, I82.411, I82.412, I82.413, I82.419, I82.42, I82.421, I82.422, I82.423, I82.429, I82.43, I82.431, I82.432, I82.433, I82.439, I82.44, I82.441, I82.442, I82.443, I82.449, I82.49, I82.491, I82.492, I82.493, I82.499, I82.4Y, I82.4Z | Acute embolism and thrombosis of deep veins of lower and upper extremity |
| Outcome | Pulmonary Embolism (PE) | ICD-10-CM: I26.01, I26.02, I26.09, I26.90, I26.92, I26.93, I26.94, I26.99 | Pulmonary embolism with and without acute cor pulmonale |
| Outcome | Blood Transfusion | CPT: 36430 | Transfusion, blood or blood components |
| Outcome | Pneumonia | ICD-10-CM: J12.0, J12.1, J12.2, J12.3, J12.81, J12.89, J12.9, J13, J14, J15.0, J15.1, J15.20, J15.211, J15.212, J15.29, J15.3, J15.4, J15.5, J15.6, J15.7, J15.8, J15.9, J16.0, J16.8, J17, J18.0, J18.1, J18.8, J18.9 | Viral, bacterial, and unspecified pneumonia |
| Outcome | Acute Respiratory Distress Syndrome (ARDS) | ICD-10-CM: J80 | Acute respiratory distress syndrome |
| Outcome | Myocardial Infarction (MI) | ICD-10-CM: I21.01, I21.02, I21.09, I21.11, I21.19, I21.21, I21.29, I21.3, I21.4, I21.9, I21.A1, I21.A9 | ST-elevation and non-ST-elevation myocardial infarction |
| Outcome | Stroke | ICD-10-CM: I63.00, I63.01, I63.02, I63.10, I63.11, I63.12, I63.20, I63.21, I63.22, I63.30, I63.31, I63.32, I63.40, I63.41, I63.42, I63.50, I63.51, I63.52, I63.6, I63.8, I63.9 | Cerebral infarction |
| Outcome | Acute Kidney Injury (AKI) | ICD-10-CM: N17.0, N17.1, N17.2, N17.8, N17.9 | Acute kidney failure |
| Outcome | Death | ICD-10-CM: R99 | Ill-defined and unknown cause of mortality; supplemented by EHR mortality flags |
| **Psychosocial Outcomes** | | | |
| Outcome | Anxiety Disorders | ICD-10-CM: F41.0, F41.1, F41.3, F41.8, F41.9 | Panic disorder, generalized anxiety disorder, other and unspecified anxiety disorders |
| Outcome | Depressive Disorders | ICD-10-CM: F32.0, F32.1, F32.2, F32.3, F32.4, F32.5, F32.81, F32.89, F32.9, F33.0, F33.1, F33.2, F33.3, F33.40, F33.41, F33.42, F33.8, F33.9 | Major depressive disorder, single episode and recurrent |
| Outcome | Opioid Use Disorder | ICD-10-CM: F11.10, F11.11, F11.12, F11.13, F11.14, F11.15, F11.18, F11.19, F11.20, F11.21, F11.22, F11.23, F11.24, F11.25, F11.28, F11.29, F11.90, F11.91, F11.92, F11.93, F11.94, F11.95, F11.98, F11.99 | Opioid abuse, dependence, and use |
| Outcome | Chronic Pain | ICD-10-CM: G89.21, G89.22, G89.28, G89.29, G89.4 | Chronic pain, not elsewhere classified |
| Outcome | Readmission | All-cause | Any inpatient admission within 365 days of index procedure |
| **Laboratory Outcomes** | | | |
| Outcome | Prothrombin Time (PT) | LOINC: 5902-2 | PT measured in seconds; reference range 11.0-13.5 seconds |
| Outcome | Activated Partial Thromboplastin Time (aPTT) | LOINC: 3173-2 | aPTT measured in seconds; reference range 25-35 seconds |
| **Matching Variables - Comorbidities** | | | |
| Matching | Diabetes Mellitus | ICD-10-CM: E08-E13 | Diabetes mellitus, all types |
| Matching | Atherosclerotic Heart Disease | ICD-10-CM: I25.10, I25.11, I25.110, I25.111, I25.118, I25.119 | Atherosclerotic heart disease of native coronary artery |
| Matching | Chronic Kidney Disease | ICD-10-CM: N18.1, N18.2, N18.30, N18.31, N18.32, N18.4, N18.5, N18.6, N18.9 | Chronic kidney disease, stages 1-5 and unspecified |
| Matching | Chronic Liver Disease | ICD-10-CM: K70, K71, K72, K73, K74, K75, K76, K77 | Diseases of liver including cirrhosis, fibrosis, hepatitis |
| Matching | COPD | ICD-10-CM: J44.0, J44.1, J44.9 | Chronic obstructive pulmonary disease |
| Matching | Peripheral Vascular Disease | ICD-10-CM: I73.0, I73.1, I73.81, I73.89, I73.9 | Peripheral vascular disease |
| Matching | Cerebrovascular Disease | ICD-10-CM: I60-I69 | Cerebrovascular diseases |
| Matching | Osteoporosis without Fracture | ICD-10-CM: M81.0, M81.6, M81.8 | Age-related and other osteoporosis without pathological fracture |
| Matching | Osteoporosis with Fracture | ICD-10-CM: M80.0, M80.8 | Age-related osteoporosis with current pathological fracture |
| Matching | Polytrauma | ICD-10-CM: T07 | Unspecified multiple injuries |
| Matching | Lower Extremity Fracture | ICD-10-CM: S82 | Fracture of lower leg, including ankle |
| **Matching Variables - Polysubstance Use** | | | |
| Matching | Alcohol-Related Disorders | ICD-10-CM: F10.10-F10.99 | Alcohol abuse, dependence, and use disorders |
| Matching | Opioid-Related Disorders | ICD-10-CM: F11.10-F11.99 | Opioid abuse, dependence, and use disorders |
| Matching | Other Psychoactive Substance Disorders | ICD-10-CM: F19.10-F19.99 | Other psychoactive substance-related disorders |
| **Matching Variables - Preoperative Antibiotics** | | | |
| Matching | Cefazolin | RxNorm: 2180 | First-generation cephalosporin |
| Matching | Vancomycin | RxNorm: 11124 | Glycopeptide antibiotic |
| Matching | Ceftriaxone | RxNorm: 2193 | Third-generation cephalosporin |
| Matching | Piperacillin/Tazobactam | RxNorm: 8339 | Extended-spectrum penicillin with beta-lactamase inhibitor |
| Matching | Gentamicin | RxNorm: 1596450 | Aminoglycoside antibiotic |
| **Fixation Procedures by Anatomical Location** | | | |
| Procedure | Shoulder/Scapula ORIF | CPT: 23515, 23585 | Open treatment of clavicular or scapular fracture |
| Procedure | Proximal Humerus ORIF | CPT: 23615, 23616, 23630 | Open treatment of proximal humeral fracture |
| Procedure | Humerus Shaft ORIF | CPT: 24515, 24516 | Open treatment of humeral shaft fracture with plate/screws |
| Procedure | Distal Humerus/Elbow ORIF | CPT: 24545, 24546, 24575, 24579, 24582, 24586, 24587 | Open treatment of humeral supracondylar, epicondylar, or condylar fracture |
| Procedure | Proximal Radius/Ulna ORIF | CPT: 24620, 24635, 24665, 24666, 24685 | Open treatment of Monteggia fracture, radial head/neck, olecranon fracture |
| Procedure | Radius/Ulna Shaft ORIF | CPT: 25515, 25525, 25526, 25545 | Open treatment of radial and/or ulnar shaft fracture |
| Procedure | Distal Radius ORIF | CPT: 25606, 25607, 25608, 25609 | Open treatment of distal radial fracture |
| Procedure | Distal Ulna ORIF | CPT: 25651, 25652 | Open treatment of ulnar styloid fracture |
| Procedure | Carpal ORIF | CPT: 25628, 25645 | Open treatment of carpal scaphoid or other carpal fracture |
| Procedure | Metacarpal ORIF | CPT: 26615, 26665 | Open treatment of metacarpal fracture |
| Procedure | Phalanx ORIF | CPT: 26735, 26746, 26765 | Open treatment of phalangeal fracture |
| **Fracture Diagnosis Codes** | | | |
| Diagnosis | Humerus Fractures | ICD-10-CM: S42.2XXA (proximal), S42.3XXA (shaft), S42.4XXA (distal) | Fracture of humerus, initial encounter for closed fracture |
| Diagnosis | Radius Fractures | ICD-10-CM: S52.1XXA (proximal), S52.3XXA (shaft), S52.5XXA (distal) | Fracture of radius, initial encounter for closed fracture |
| Diagnosis | Ulna Fractures | ICD-10-CM: S52.0XXA (proximal), S52.2XXA (shaft), S52.6XXA (distal) | Fracture of ulna, initial encounter for closed fracture |
| Diagnosis | Hand/Wrist Fractures | ICD-10-CM: S62.0XXA-S62.6XXA | Fracture of navicular, other carpal, metacarpal, and phalanges |
| Diagnosis | Open Fracture Modifier | 7th character B or C | B = open fracture type I/II, C = open fracture type IIIA/B/C |

**Supplemental Table 2 – Post-Matching Surgical Management Characteristics**

|  | **Cannabis-Only Users vs Non-Users (N=801)** | | | **Nicotine-Only Users vs Non-Users (N=14,310)** | | | **Concurrent Users vs Cannabis-Only Users (N=901)** | | |
| --- | --- | --- | --- | --- | --- | --- | --- | --- | --- |
| **Characteristic** | **Exposed** | **Control** | **SMD** | **Exposed** | **Control** | **SMD** | **Exposed** | **Control** | **SMD** |
| **Fracture Location** | | | | | | | | | |
| Hand/Wrist | 346 (36.5%) | 371 (39.1%) | 0.054 | 5552 (35.0%) | 6005 (37.9%) | 0.059 | 335 (36.8%) | 341 (37.4%) | 0.014 |
| Proximal Humerus | 101 (10.6%) | 113 (11.9%) | 0.040 | 2330 (14.7%) | 2505 (15.8%) | 0.031 | 96 (10.5%) | 101 (11.0%) | 0.018 |
| Humerus Shaft | 172 (18.1%) | 192 (20.2%) | 0.054 | 2505 (15.8%) | 2646 (16.7%) | 0.024 | 151 (16.5%) | 167 (18.2%) | 0.046 |
| Distal Humerus | 138 (14.5%) | 134 (14.1%) | 0.012 | 2136 (13.5%) | 2073 (13.1%) | 0.012 | 124 (13.5%) | 127 (13.9%) | 0.010 |
| Forearm (Radius/Ulna) | 268 (28.2%) | 193 (20.3%) | 0.185 | 3444 (21.7%) | 3735 (23.6%) | 0.044 | 268 (29.2%) | 268 (29.2%) | 0.000 |
| **Fracture Type** | | | | | | | | | |
| Closed Fracture | 426 (44.9%) | 418 (44.0%) | 0.017 | 7974 (50.3%) | 8466 (53.4%) | 0.062 | 393 (42.9%) | 411 (44.8%) | 0.040 |
| Open Fracture | 191 (20.1%) | 134 (14.1%) | 0.160 | 1011 (6.4%) | 1007 (6.4%) | 0.001 | 195 (21.3%) | 191 (20.8%) | 0.011 |
| **Injury Characteristics** | | | | | | | | | |
| Polytrauma | 81 (8.5%) | 69 (7.3%) | 0.047 | 1136 (7.2%) | 1176 (7.4%) | 0.010 | 88 (9.6%) | 81 (8.8%) | 0.026 |
| **Preoperative Antibiotics** | | | | | | | | | |
| Cefazolin | 401 (42.3%) | 401 (42.3%) | 0.000 | 5632 (35.5%) | 6026 (38.0%) | 0.052 | 401 (43.7%) | 395 (43.1%) | 0.013 |
| Vancomycin | 105 (11.1%) | 101 (10.6%) | 0.014 | 1614 (10.2%) | 1659 (10.5%) | 0.009 | 108 (11.8%) | 105 (11.5%) | 0.010 |
| Ceftriaxone | 81 (8.5%) | 83 (8.7%) | 0.008 | 1568 (9.9%) | 1582 (10.0%) | 0.003 | 82 (8.9%) | 83 (9.1%) | 0.004 |
| Piperacillin | 51 (5.4%) | 58 (6.1%) | 0.032 | 1002 (6.3%) | 981 (6.2%) | 0.005 | 48 (5.2%) | 52 (5.7%) | 0.019 |
| Gentamicin | 42 (4.4%) | 40 (4.2%) | 0.010 | 581 (3.7%) | 613 (3.9%) | 0.011 | 47 (5.1%) | 42 (4.6%) | 0.025 |
| **Fixation Type** | | | | | | | | | |
| Proximal Humerus ORIF | 10 (1.1%) | 10 (1.1%) | 0.000 | 26 (0.2%) | 22 (0.1%) | 0.006 | 0 (0%) | 10 (1.1%) | 0.148 |
| Humerus Shaft ORIF | 10 (1.1%) | 0 (0%) | 0.146 | 25 (0.2%) | 13 (0.1%) | 0.022 | 10 (1.1%) | 10 (1.1%) | 0.000 |
| Supracondylar Humerus ORIF | 0 (0%) | 10 (1.1%) | 0.146 | 13 (0.1%) | 11 (0.1%) | 0.005 | 10 (1.1%) | 0 (0%) | 0.148 |
| Proximal Ulna ORIF | 10 (1.1%) | 10 (1.1%) | 0.000 | 40 (0.3%) | 29 (0.2%) | 0.015 | 10 (1.1%) | 10 (1.1%) | 0.000 |
| Radius/Ulna Shaft ORIF | 10 (1.1%) | 10 (1.1%) | 0.000 | 39 (0.2%) | 19 (0.1%) | 0.030 | 10 (1.1%) | 10 (1.1%) | 0.000 |
| Distal Radius ORIF | 10 (1.1%) | 10 (1.1%) | 0.000 | 109 (0.7%) | 100 (0.6%) | 0.007 | 10 (1.1%) | 10 (1.1%) | 0.000 |
| Metacarpal ORIF | 10 (1.1%) | 0 (0%) | 0.146 | 33 (0.2%) | 18 (0.1%) | 0.024 | 10 (1.1%) | 10 (1.1%) | 0.000 |
